# Preoperative fasting in noncardiac surgery in a tertiary pediatric center applying European guidelines: the difficulty of giving drink to the thirsty

**DOI:** 10.1186/s44158-025-00247-w

**Published:** 2025-05-16

**Authors:** Zaccaria Ricci, Denise Colosimo, Francesca Donati, Luca Saccarelli, Mariateresa Pizzo, Elena Schirru, Salvatore Giacalone, Paola Serio

**Affiliations:** 1 Anesthesiology Unit, Department of Anesthesia and Critical Care, Meyer Children’s Hospital, IRCCS, Florence, Italy; 2 Section of Anesthesiology and Intensive Care, Department of Health Sciences, University of Florence, Florence, Italy; 3 Neuro-Anesthesiology Unit, Department of Anesthesia and Critical Care, Meyer Children’s Hospital, IRCCS, Florence, Italy; 4 Pediatric Intensive Care Unit, Viale Pieraccini 24, 50139 Florence, Italy

**Keywords:** Preoperative fasting, Clear fluids, Non-clear fluids, Hypotension, Pediatric surgery

## Abstract

**Background:**

We conducted a secondary analysis of a previously published dataset that addressed clear fluid fasting in children. The aim of this single-center, retrospective observational study conducted in a tertiary level pediatric hospital (Meyer Children’s Hospital, Florence, Italy) was to report updated results after enrollment of new patients, including clear and non-clear fluids and meals.

**Methods:**

Retrospective single-center study in a tertiary pediatric hospital after the implementation of an improvement bundle.

**Results:**

Overall, we enrolled 2715 patients, and after exclusion of 199 children due to incomplete data retrieval, a final cohort of 2516 subjects (1074 surgical outpatients, 981 surgical inpatients, 314 neurosurgical procedures, 147 procedures from pediatrics/oncology) was analyzed. Median age was 7.5 (3.7–12.2) years. Median (interquartile range) preoperative fasting time was 187 (119–351) min for clear fluids, 286 (218–396) min for maternal milk, 360 (285–530) min for artificial milk, 435 (350–540) min for light breakfast, and 765 (640–910) min for meal. We did not find significant differences between the clear fluid times of the previous study (1820 patients, 185 (115–340) min) and the one analyzed in the present dataset (696 patients, 192 (120–363) min) (*p* = 0.12).

**Conclusion:**

In a pediatric hospital implementing European Society of Anesthesia recommendations for preoperative fasting, all fluids and meals are stopped very far from the scheduled times, and this notion should provide ignition for further improvement actions.

**Supplementary Information:**

The online version contains supplementary material available at 10.1186/s44158-025-00247-w.

Dear Editor

We conducted a secondary analysis of a previously published dataset that addressed clear fluid fasting in children conducted from January to June 2023 [1]. The aim of this new single-center retrospective observational study conducted in a tertiary level pediatric hospital (Meyer Children’s Hospital, Florence, Italy) was to report updated results after the enrollment of new patients from October to December 2024. Local Pediatric Ethics Committee approval was obtained, with the exemption from informed consent due to the design of the study (Comitato Etico Regionale per la Sperimentazione Clinica della Regione Toscana, Sezione: COMITATO ETICO PEDIATRICO). The study was conducted in accordance with the Declaration of Helsinki.

An improvement bundle to reduce fasting times was planned after auditing the data from the first study (supplement). In this update, we inform about the results of this action and add information about all non-clear fluids and meals. Institutional protocol for fasting times has been previously described [1].

Overall, we screened 2715 patients, and, after exclusion of 199 children due to incomplete data, we enrolled a final cohort of 2516 subjects (1820 patients in the first period and 696 in the second), of which 1074 surgical outpatients, 981 surgical inpatients, 314 neurosurgical procedures, and 147 were procedures from pediatrics/oncology. The median age was 7.5 (3.7–12.2) years. Fasting times are described in Table 1: the median (interquartile range) preoperative fasting time was 187 (119–351) min for clear fluids, 286 (218–396) min for maternal milk, 360 (285–530) min for artificial milk, 435 (350–540) min for light breakfast, and 765 (640–910) min for a meal. We did not find significant differences between the clear fluid times of the previous study, 185 (115–340) min, and the one analyzed in the present dataset, 192 (120–363) min (*p* = 0.12). We confirm that the type of surgery predicted the capacity for shortening fasting times, with major surgeries performing better than outpatient surgery (Table 2) since they likely allow a less uncertain operating theatre schedule and more precise times of surgery. Reported adverse events (hypotension and tachycardia at induction, vein cannulation after multiple attempts) were 98 in the first period (5.4%) and 39 in the second (5.6%) (*p* = 0.59).

**Table 1:** Fasting times: median and interquartile range (IQR) of clear and non-clear fluids and meals (*MIN*, minutes)

Fasting MIN	Median	(IQR)
Clear	185	(115–340)
Maternal milk	286	(218–396)
Artificial milk	360	(285–530)
Light breakfast	435	(350–540)
Meal	765	(640–910)

**Table 2:** Fasting times stratified on different admission wards

Minutes	**Outpatients**	**Surgery**	**Neurosurgery**	**N-S wards**	***p***
Clear	248 (140–568)	163 (105–269)^a^	120 (90–190)^a^	200 (120–342)^a^	< 0.0001
Maternal milk	295 (230–453)	300 (245–435)	230 (195–284)^a^	315 (213–483)	0.0021
Artificial milk	504 (348–738)	385 (310–504)	285 (269–380)^a^	360 (297–677)	0.0003
Light breakfast	427 (265–495)	460 (375–560)	370 (289–435)	400 (321–567)	0.0004
Meal	780 (706–895)	760 (580–960)	660 (500–780)^a^	689 (502–934)^a^	< 0.0001

*N-S* non-surgical

^a^Significantly different vs. outpatients (*p* < 0.01) at post hoc test

Our cohort of children scheduled for elective surgery confirmed that times of clear, non-clear fluids and meal fasting are far from protocol, in line with recent literature [2–4]. Our findings remark that in light of the adoption of a liberal protocol for fasting before surgery, times for clear fluids remain around 3 h and for meals around 10 h. A strong resistance to shorten non-clear fluids and meal fasting is evident from this investigation, which came after an attempt to improve communication and the introduction of strategies to implement drinking. The source of resistance remained in 40% of cases due to organizational issues (uncertainty of the exact time of surgery scheduling) and in 60% of cases due to patients’ related concerns (they did not want to drink, the parents did not understand the instructions, or they were unsure about the times and afraid of rescheduling). In our opinion, the most effective way of managing organizational issues might be to apply the “Sip Til Send” protocol [5] that would exclude any need for “guessing” the exact surgery time. Patient-related fasting noncompliance is more difficult to manage and should be driven by a cultural shift throughout all the communications during the surgical pathway: this topic should be properly addressed in all the pre-surgery interactions with parents and patients (i.e., surgical lab, anesthesiology lab, hospital admission) in order to consistently educate them and eventually minimize the unwanted side effects of prolonged fasting (dehydration, thirst, irritability, metabolic consequences).

In conclusion, in a pediatric hospital implementing European Society of Anesthesia recommendations for preoperative fasting [6], all fluids and meals are stopped very far from the scheduled times, and this notion should provide ignition for more efficient improvement actions.

## Supplementary Information

Additional file 1: Supplement. Bundle for improving fasting times at our institution (decided on June 2025 and conducted from September to December 2024).

## Data Availability

No datasets were generated or analysed during the current study.

## References

[1] Ricci Z, Colosimo D, Saccarelli L et al (2024) Preoperative clear fluids fasting times in children: retrospective analysis of actual times and complications after the implementation of 1-h clear fasting. J Anesth Analg Crit Care 4(1):12 38350987 10.1186/s44158-024-00149-3 PMC10865513

[2] Aroonpruksakul N, Punchuklang W, Kasikan K, Laotaweesuk N, Phoson P, Khongrod R, Kiatchai T (2023) The actual duration of preoperative fasting in pediatric patients, and its effects on hunger and thirst: a prospective observational study. Transl Pediatr 12(2):146–154 36891367 10.21037/tp-22-358 PMC9986785

[3] Webb AR, Kalam I, Lui N, Loughnan RM, Leong S (2024) A pre and post interventional audit of an ‘apple juice on arrival’ protocol to reduce excessive clear fluid fasting times in paediatric patients. Anaesth Intensive Care 52:328–334 39212180 10.1177/0310057X241263112

[4] de Klerk ES, de Grunt MN, Hollmann MW, Preckel B, Hermanides J, van Stijn MFM (2023) Incidence of excessive preoperative fasting: a prospective observational study. Br J Anaesth 130:e440–e442 36670008 10.1016/j.bja.2022.12.017

[5] Harnett C, Connors J, Kelly S, Tan T, Howle R (2024) Evaluation of the ‘Sip Til Send’ regimen before elective caesarean delivery using bedside gastric ultrasound: a paired cohort pragmatic study. Eur J Anaesthesiol 41(2):129–135 37982593 10.1097/EJA.0000000000001926

[6] Frykholm P, Disma N, Andersson H, Beck C, Bouvet L, Cercueil E et al (2022) Pre-operative fasting in children: a guideline from the European Society of Anaesthesiology and Intensive Care. Eur J Anaesthesiol 39:4–25 34857683 10.1097/EJA.0000000000001599

