# Supplementary material for: Preoperative fasting in noncardiac surgery in a tertiary pediatric center applying European guidelines: the difficulty of giving drink to the thirsty
Source: J Anesth Analg Crit Care. 2025 May 16;5:27. doi: 10.1186/s44158-025-00247-w (PMC12085058; doi:10.1186/s44158-025-00247-w)
Supplement: Supplementary file 1 — Additional file 1: Supplement. Bundle for improving fasting times at our institution (decided on June 2025 and conducted from September to December 2024). [file 44158_2025_247_MOESM1_ESM.docx]

**Bundle for improving fasting times at our institution (decided on June 2025 and conducted from September to December 2024):**

1) the preoperative informative brochure has been modified to better evidence the clear fasting rules description

2) at patient hospital admission the notions of fasting and the local protocol need to be repeated by the acceptance personnel to all arriving families, in order to be sure that they have been clearly comprised the importance of appropriate fasting times

3) on the day of scheduled surgery, the attending anesthesiologists need to confirm to the ward-attending nurse, before the first patient is called, all the due clear fluids fasting times of the listed children. An anesthesiologist is also available for updates on schedule modifications

Monitoring of the effects of the present bundle was made from October 2024 to December 2024.
